# Ovarian Cancer Treatments Strategy: Focus on PARP Inhibitors and Immune Check Point Inhibitors

**DOI:** 10.3390/cancers13061298

**Published:** 2021-03-15

**Authors:** Camilla Nero, Francesca Ciccarone, Antonella Pietragalla, Simona Duranti, Gennaro Daniele, Vanda Salutari, Maria Vittoria Carbone, Giovanni Scambia, Domenica Lorusso

**Affiliations:** 1 Fondazione Policlinico Universitario A. Gemelli IRCCS, Direzione Scientifica, 00165 Roma, Italy; francesca.ciccarone@policlinicogemelli.it (F.C.); Antonella.pietragalla@policlinicogemelli.it (A.P.); simona.duranti@policlinicogemelli.it (S.D.); gennaro.daniele@policlinicogemelli.it (G.D.); giovanni.scambia@policlinicogemelli.it (G.S.); domenica.lorusso@policlinicogemelli.it (D.L.); 2 Department of Obstetrics and Gynecology, Università Cattolica del Sacro Cuore, 00165 Roma, Italy; 3 Fondazione Policlinico Universitario A. Gemelli IRCCS, Dipartimento per le Scienze Della Salute Della Donna, del Bambino e di Sanità Pubblica, 00165 Roma, Italy; vanda.salutari@policlinicogemelli.it (V.S.); mariavittoria.carbone@guest.policlinicogemelli.it (M.V.C.)

**Keywords:** ovarian cancer, PARP inhibitors, immunotherapy, targeted therapy

## Abstract

**Simple Summary:**

Ovarian cancer treatment is the deadliest of gynecological cancers but the introduction of target therapies in its therapeutic algorithm has significantly improved outcomes. The aim of this article was to provide a review of completed and ongoing clinical trials involving the use of PARP inhibitors and/or immune checkpoint inhibitors for the treatment of ovarian cancer in front line and relapse settings. We confirmed the role of bevacizumab and PARP inhibitors which have transformed OC into a chronic disease while almost all trials involving immunotherapy were disappointing.

**Abstract:**

Ovarian cancer treatment strategy is mainly based on three pillars: cytoreductive surgery, platinum-based chemotherapy, and targeted therapies. The latter in the last decade has provided a remarkable improvement in progression free patients and, hopefully, in overall survival. In particular, poly(adenosine diphosphate-ribose) polymerase (PARP) inhibitors exploit BRCA 1/2 mutations and DNA damage response deficiencies, which are believed to concern up to 50% of high grade epithelial ovarian cancer cases. While these agents have an established role in ovarian cancer treatment strategy in BRCA mutated and homologous recombination deficient patients, an appropriate predictive molecular test to select patients is lacking in clinical practice. At the same time, the impressive results of immunotherapy in other malignancies, have opened the space for the introduction of immune-stimulatory drugs in ovarian cancer. Despite immune checkpoint inhibitors as a monotherapy bringing only modest efficacy when assessed in pretreated ovarian cancer patients, the combination with chemotherapy, anti-angiogenetics, PARP inhibitors, and radiotherapy is believed to warrant further investigation. We reviewed literature evidence on PARP inhibitors and immunotherapy in ovarian cancer treatment.

## 1. Introduction

Ovarian cancer (OC) treatment strategy has dramatically changed over the last decade.

Until 2011, OC treatment was based on cytoreductive surgery and platinum-based chemotherapy. Despite the evolution of surgical techniques and chemotherapy treatment schedules, prognostic improvements were minimal and relapse remained almost inevitable in most patients with advanced disease [1].

The advent of targeted therapies transformed the OC treatment landscape, both in the first line and in the recurrent clinical setting [2,3,4,5].

The established targeted agents in the OC therapeutic algorithm are vascular endothelial growth factor (VEGF) and poly(adenosine diphosphate-ribose) polymerase (PARP) inhibitors.

Despite the positive outcome results achieved in clinical trials, there remain several challenges to further refine their optimal positioning in OC treatment algorithms.

In particular, the identification of the correct population to treat and the optimal time window to prescribe, as well as a better understanding of mechanisms underlying drug resistance, are crucial points to be addressed.

Useful, although not validated, considerations for treatment approach can be developed mainly based on inference from analyzed subgroups in clinical trials [6,7].

**Figure 1:**
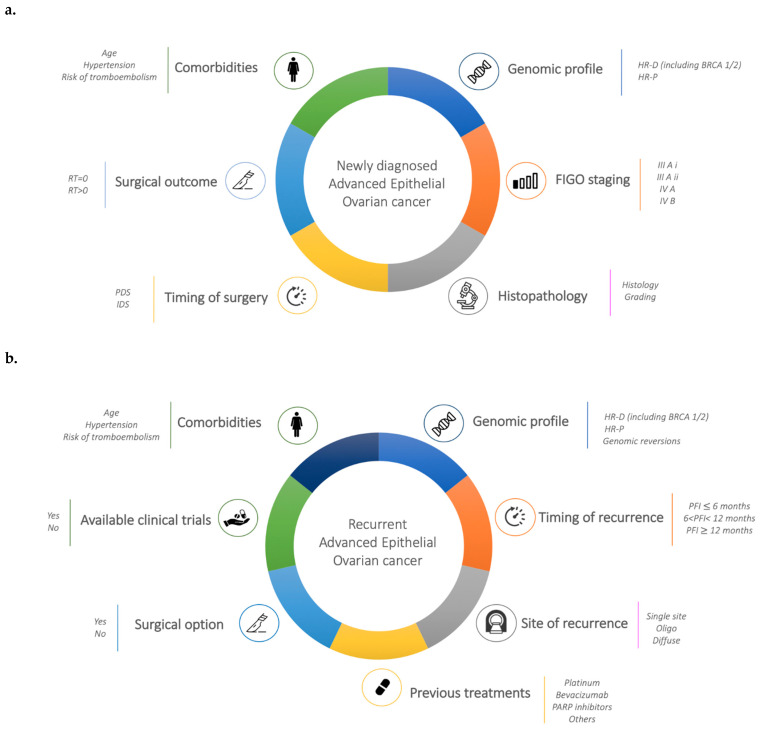
(**a**). Determinants of forefront ovarian cancer (OC) therapeutic strategy. (**b**). Determinants of recurrent OC therapeutic strategy.

The main determinants of forefront and recurrent OC therapeutic strategy are summarized in Figure 1a,b. Several considerations should be taken into account when choosing first line and second line treatments: patients and disease characteristics may play a role in determining the choice. In particular, patient comorbidities, patient preference, tumor stage, residual disease after surgery, and toxicity profile of the drug, as well as residual toxicity at previous treatments and molecular characteristic of tumor are all aspects that may influence treatment decision and that need to be carefully addressed.

As far as bevacizumab is concerned, there is no unanimous agreement on its prescription in clinical practice in front-line maintenance treatment, given the absence of validated predictive molecular biomarkers of response, the lack of evidence of OS advantage in the overall population and in low risk patients in particular, and the better hazard ratio (HR) reported in the second line settings [5].

Regarding PARP-inhibitors, the lack of an appropriate test to determine sensitivity to these agents, besides BRCA mutation, prevents maximizing their effect. Current assays of homologous recombination deficiency (HRD), in fact, are not so sensitive to effectively exclude patients from PARP inhibitor therapy [8].

The spotlight is now on the role of immunotherapy (IO) in OC treatment in combination with chemotherapy and/or with other biological agents. Although no immunotherapy agent is yet approved in OC, due to the disappointing results in clinical trials, there is still a lot of expectation. Synergistic effects with other biological agents and/or the identification of a predictive biomarker of response to IO are believed to positively change the perception of IO in OC.

In this article, we reviewed the state of the art and the most relevant perspectives of targeted therapies in OC, focusing on PARP inhibitors and immune check point inhibitors.

## 2. State of the Art Treatment of Newly Diagnosed Ovarian Cancer

**Figure 2:**
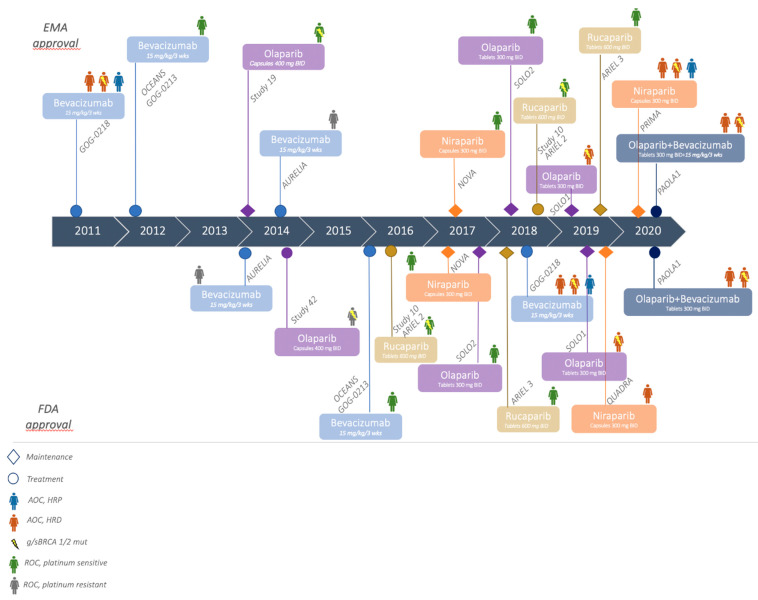
A timeline of targeted therapies approvals in OC by both the Food and Drug Administration (FDA) and European Medicines Agency (EMA).

A timeline of targeted therapy approvals by both the Food and Drug Administration (FDA) and The European Medicines Agency (EMA) in all OC clinical settings, is illustrated in Figure 2.

**Figure 3:**
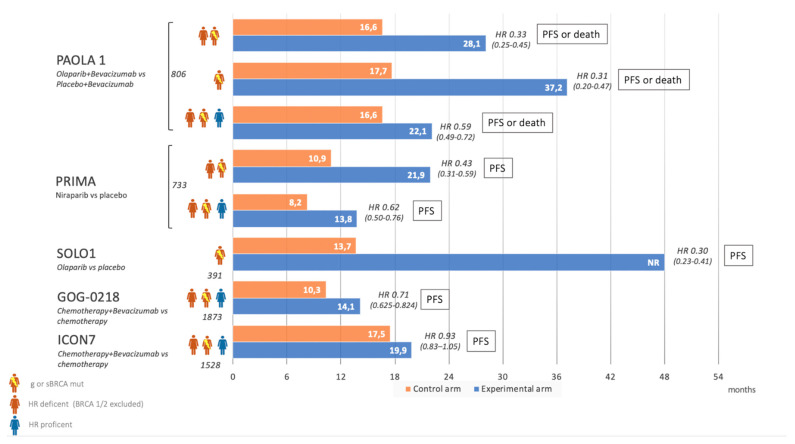
Clinical trials in front line OC treatment: median progression-free survival (PFS) of approved agents.

Carboplatin-paclitaxel IV every 3 weeks for 6 cycles is the standard of care in newly diagnosed ovarian cancer. In this clinical setting, the FDA and EMA recommended granting a marketing authorization for bevacizumab, olaparib, the combination of olaparib and bevacizumab, and niraparib as maintenance treatments. Figure 3 illustrates the median progression-free survival (PFS) of approved agents considering data from clinical trials.

### 2.1. Bevacizumab

Bevacizumab is approved for advanced newly diagnosed OC patients in combination with chemotherapy and as maintenance for 15 months, based on the progression-free survival (PFS) benefit observed in the GOG-0218 (∆ PFS 3.8 months, HR 0.75) and ICON7 (∆ PFS 2,4 months, HR *0.93*) trials [9,10]. Given the absence of OS advantage in the overall population and the limited benefit in terms of PFS, it has been debated whether it should be restricted only to high-risk patients (stage IV disease or stage III with macroscopic residual disease after primary debulking surgery) given the OS advantage reported by post-hoc subgroup analysis, and also considering the toxicity profile of the drug and the risk benefit balance for OC patients. [11,12,13]. No differences in terms of quality of life were reported in GOG-218, while a minimal deterioration in quality of life was reported in the ICON 7 trial, possibly due to the absence of active treatment in the control arm, which determined period life interruption only in patients receiving Bevacizumab.

### 2.2. Role of Parp Inhibitor in First Line Treatment: Olaparib

Olaparib received authorization as a monotherapy for the maintenance treatment of adult patients with advanced (FIGO stages III and IV) BRCA1/2-mutated (germline and/or somatic) high-grade epithelial OC patients who are in response (complete or partial) following completion of first-line platinum-based chemotherapy, and based on the PFS benefit observed in the SOLO 1 trial (PFS in experimental arm approximately 3 years longer than the placebo group, HR *0.30*) [14]. In the experimental arm olaparib was given at 300 mg BID for up to 2 years. The most common grade ≥3 adverse event (AE) reported in the olaparib arm was anemia (7%). At present, no mature OS data are available, while quality of life did not report any difference between the two treatment arms.

The two major limitations to the general applicability of this trial are the restriction to BRCA 1/2 mutated patients and the lack of bevacizumab containing therapy in the control arm.

### 2.3. Olaparib and Bevacizumab Combination

The combination of olaparib and bevacizumab was recently approved as a maintenance treatment of adult patients with advanced (FIGO stages III and IV) high-grade, HRD positive, epithelial OC who are in response (complete or partial) following completion of first-line platinum-based chemotherapy.

This indication was based on the results coming from the PAOLA 1 trial, a randomized phase III study comparing bevacizumab alone (standard schedule) or bevacizumab plus olaparib (300 mg BID) for 24 months in high grade serous and endometroid OC patients completing carboplatin–paclitaxel–bevacizumab chemotherapy in partial or complete response [15]. The molecular test used to assess HRD status was the Myriad MyChoiceHRD^®^ (Myriad Genetic Laboratories, Inc., Salt Lake City, UT, USA). The test evaluates the presence of loss of heterozygosity (LOH), telomeric allelic imbalance, and large-scale state transitions across the genome, and the readout of this assay is presented as an ‘HRD score’; a tumor with an HRD score ≥42 is labelled as HRD-positive [16].

Overall, the advantage in terms of PFS obtained by the experimental arm was 5.5 months (HR = *0.59*). The maximum benefit however was observed in the BRCA mutant population (∆ PFS 19.5 months, HR = *0.31*) and in the HRD patients without BRCA mutations (∆ PFS 11.5 months, HR = *0.33*). At present, no mature OS data are available, while quality of life did not report any difference between the two treatment arms.

The major limitation of this trial was the absence of an olaparib alone arm, as it is not possible to determine the contribution of bevacizumab to the PARP single agent activity. Moreover, it excluded patients considered ineligible to bevacizumab.

Overall, an increased incidence of hypertension (19% vs. 30%) and anemia (17% vs. <1%) was observed in the combination arm. When considering the molecular subgroups enrolled in the trial, it is debated if BRCA mutated patients should receive the combination of bevacizumab + olaparib, considering the outstanding results of olaparib single agent in BRCA mutated patients (48% patients free from recurrence at 5 year follow up), thus questioning what is the added benefit of bevacizumab in this population that justifies the additional toxicity.

### 2.4. Niraparib

Niraparib received authorization as a monotherapy for the maintenance treatment of adult patients with advanced epithelial (FIGO Stages III and IV) high-grade OC who are in response (complete or partial) following completion of first-line platinum-based chemotherapy.

Evidence for this indication came from the PRIMA/ENGOT-OV26 study, a randomized phase III trial comparing niraparib (300 mg/once daily) for up to 3 years versus placebo in patients with disease at high risk of treatment failure (RT > 0, stage IV) [17]. All the patients were tested for homologous recombination deficiency (HRD) through Myriad MychoiceHRD^®^, with ≥42 identified as the cutoff for positivity.

Among the HRD patients, the advantage in terms of PFS obtained by the experimental arm was 11 months (HR *0.43*), while it was 5.6 months (HR *0.62*) in the overall population. At present, no mature OS data are available, while quality of life did not report any difference between the two treatment arms.

The study has two main limitations: the lack of bevacizumab in the control arm, particularly in the clinical setting, in which it could be considered the standard of care (high risk patients), and the exclusion of optimally debulked patients at primary surgery, who should be a non-negligible number in referral centers. Moreover, the limited PFS benefit in HR proficient patients questions what is the best treatment for this population; bevacizumab or niraparib. Given the absence of a head to head comparison between the two treatments, at the present moment we believe the choice of treatment should be discussed with the patient and tailored according to the toxicity profile of the drug. As a general consideration, PARP inhibitor treatment, which is linked to platinum response, should be anticipated as early as possible in the treatment algorithm of patients, given that the response to platinum tends to decrease over time.

The most commonly reported grade ≥3 AEs were anemia (31%), thrombocytopenia (29%), and neutropenia (13%).

## 3. State of the Art Treatment in Relapse Ovarian Cancer

**Figure 4:**
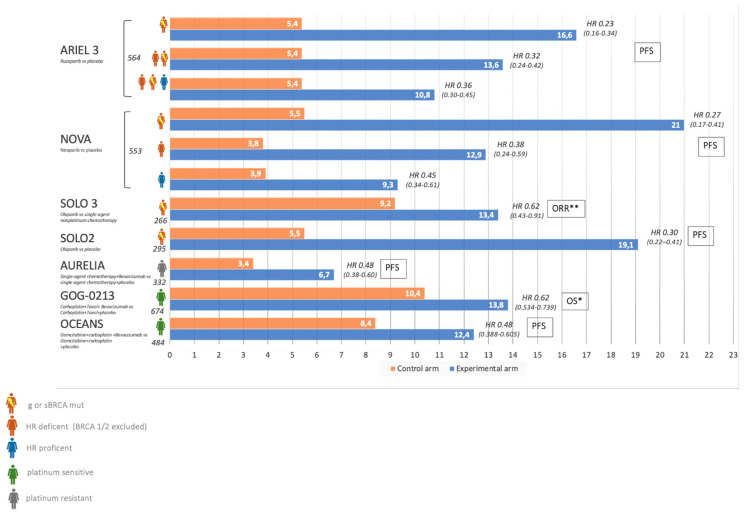
Recurrent OC treatment: median PFS from clinical trials which lead to agent approval. *OS was the primary endpoint, but months reported express PFS for all trials in the figure. **ORR: Objective response rate.

In this clinical setting, the FDA and EMA recommended granting a marketing authorization for bevacizumab, olaparib, niraparib, and rucaparib. Figure 4 illustrates the median PFS of the approved agents considering data from the clinical trials. At present time, all these drugs can be prescribed once during the natural history of disease, and none of them are being reimbursed, in Europe, beyond progression. The choice of second line treatment is primarily influenced by what kind of treatment patients received in the first line. Moreover, the toxicity profile of the drug, clinical characteristics (ascites, tumor burden), and residual toxicity should be taken into consideration when choosing the target agent. Finally, genomic profile is an important issue in the choice of treatment, and BRCA mutant patients who have not received PARP inhibitor in the first line should be treated as early as possible with PARP at the time of recurrence.

### 3.1. Bevacizumab

Bevacizumab is indicated in combination with platinum-base chemotherapy for the treatment of adult patients with platinum-sensitive epithelial OC recurrence, and who have not received prior therapy with bevacizumab or other VEGF inhibitors or VEGF receptor-targeted agents. Evidence for this indication came from the PFS advantage registered in the OCEANS (∆ PFS 4 months, HR *0.48*) and GOG-0213 (∆ PFS 3,4 months, HR *0.63*) trials [18,19]. The primary end point of GOG-0213 was OS; a very close to significant OS advantage was reported in the trial (HR *0.829*; 95% CI 0.683–1·005; *p* = 0.056), while no statistically significant OS advantage was reported in the OCEANS trial. No deterioration in terms of quality of life was reported in both trials between patients receiving bevacizumab or not.

Moreover, it is indicated for the treatment of adult patients with platinum-resistant recurrent epithelial ovarian, fallopian tube, or primary peritoneal cancer who received no more than two prior chemotherapy regimens, and who have not received prior therapy with bevacizumab or other VEGF inhibitors in combination with paclitaxel, topotecan, or pegylated liposomal doxorubicin, and based on the PFS advantage identified in the AURELIA trial (∆ PFS 3,3, HR *0.48*) [20]. No OS advantage was reported in the overall population; post hoc subgroup analysis reported a 11-month increase in OS in patients receiving weekly paclitaxel + beva with respect to weekly paclitaxel alone. A significant amelioration in quality of life was reported in patients receiving bevacizumab, particularly in terms of gastrointestinal symptoms and ascites.

### 3.2. Role of Parp Inhibitors in Relapse Disease: Olaparib

Olaparib is currently indicated as a monotherapy for the maintenance treatment of patients with platinum-sensitive relapsed high-grade epithelial OC who are in complete (CR) or partial response (PR) to platinum-based chemotherapy. On August 2017 and May 2018, the FDA and EMA respectively, extended olaparib indication irrespective of BRCA status. The evidence supporting these indications came from four clinical trials.

Overall, the advantage in terms of PFS obtained by the experimental arm was 3.6 months (HR *0.35*); in the BRCA1/2 (somatic and germline) mutated patients the magnitude of PFS benefit was more evident (∆ PFS 6.9 months, HR *0.18*). However, no significant difference in terms of overall survival (OS) was observed in the overall population and in BRCA mutated patients (29.8 vs. 27.8 months, HR 0.73, and 34.9 vs. 30.2 months, HR *0.62*, for olaparib and placebo, respectively).

The SOLO3 (NCT02282020) trial was a randomized phase III trial on 266 gBRCA mutated platinum sensitive relapsed OC patients, comparing olaparib tablets (300 mg BID) versus non platinum treatment (weekly paclitaxel, weekly topotecan, gemcitabine, or pegylated liposomal doxorubicin) [21]. The overall response rate (72% vs. 51%, OR 2.53, *p* = 0.002) and the PFS (median 13.4 vs. 9.2 months, HR= *0.62*) favored Olaparib. At present, no mature OS data are available, while quality of life did not report any difference between the two treatment arms.

SOLO2/ENGOT-Ov21 trial, was a phase III trial on 295 platinum sensitive, recurrent BRCA1/2 mutated OC patients designed to assess the efficacy and safety of olaparib (300 mg twice daily) versus placebo [22] as maintenance after CR or PR to platinum-containing chemotherapy.

The advantage in terms of PFS obtained by olaparib was 13.6 months (HR *0.30*).

Overall, nausea, fatigue, vomiting, and anemia, primarily of grade 1 or 2, were the adverse effects most frequently reported in olaparib users across all the above studies.

A recent update of OS data reported a clinically significant 13-month increase in OS. The advantage was not statistically significant, possibly due to the high rate (38%) of crossover of patients receiving PARP inhibitors after progression in the placebo arm. No detrimental effect of Olaparib on quality of life was reported.

### 3.3. Niraparib

Niraparib is currently indicated as a monotherapy for the maintenance treatment of adult patients with platinum-sensitive relapsed high grade serous epithelial OC who are in response (complete or partial) to platinum-based chemotherapy.

Data supporting this indication came from the ENGOTOV16/NOVA trial, a randomized phase III study on 553 relapsed platinum sensitive OC patients (203 with germline (g)BRCA mutation and 350 without gBRCA mutation) treated with at least two previous platinum-based regimens, and aimed at comparing niraparib (300 mg/day) with placebo [23]. Germline BRCA status was assessed with Myriad Genetics myChoice HRD test. The advantage in terms of PFS obtained by the experimental arm was 15.5 months (HR *0.27*) in the gBRCA mutated population, 5.4 months in the gBRCA group WT, and 9.1 month (HR *0.38*) in the HRD-positive cohort compared to the placebo group. Overall, grade 3–4 thrombocytopenia, anemia, and neutropenia were the adverse effects most frequently reported in Niraparib users (34%, 25%, and 20% respectively). At present, no mature OS data are available, while quality of life did not report any difference between the two treatment arms.

### 3.4. Rucaparib

Rucaparib is indicated as a monotherapy both for the maintenance treatment of patients with platinum-sensitive relapsed high-grade epithelial OC who are in response (complete or partial) to platinum-based chemotherapy and for the treatment of platinum sensitive, relapsed or progressive, BRCA mutated (germline and/or somatic), high-grade epithelial OC, who have been treated with two or more prior lines of platinum based chemotherapy, and who are unable to tolerate further platinum-based treatments.

Evidence on such indications was drawn from two clinical trials.

ARIEL2- part 1 (NCT01891344) was a single arm, phase II trial on 204 relapsed high grade serous or endometrioid OC patients treated with at least one prior platinum containing regimen, and aimed at evaluating the activity of rucaparib (600 mg BID) as single agent [24].

HRD was assed using FoundationFocus™ CDx BRCA _LOH_; the test detects the presence of mutations in the BRCA1/2 genes and the percentage of the genome affected by loss of heterozygosity (LOH) in DNA from tumor tissue samples [8].

In this trial the tumors were categorized as LOH-high if the score was ≥16%.

Mean PFS after rucaparib treatment was 12.8 months in the BRCA mutated group, 5.7 months in the LOH-high group, and 5.2 months in the LOH-low group. To assess the HRD status, the FDA simultaneously approved FoundationFocus™ CDx BRCA LOH as a companion diagnostic test.

The ARIEL3 trial (NCT01968213) was a randomized phase III study on 564 OC patients previously treated with at least two prior platinum-based chemotherapy lines, and aiming at comparing rucaparib (600 mg oral BID) or placebo as a maintenance treatment after CR or PR to last platinum-based chemotherapy.

A hierarchical statistical design was implemented, with the results analyzed first in BRCA mutated patients, then in an HRD population, and finally in the intention to treat population. The HRD status was assessed by the FoundationFocus™ CDx BRCA LOH test.

The advantage in terms of PFS obtained by the experimental arm was 5.4 months (HR 0.36) in the overall population, 11.2 months (HR *0.23*) for BRCA-mutated patients, and 8.2 months (HR *0.32*) in the HRD-positive population.

Grade 3/4 anemia (19%) and an increase in alanine or aspartate aminotransferase (10%) were the adverse effects most frequently reported in rucaparib users. At present, no mature OS data are available, while quality of life did not report any difference between the two treatment arms.

## 4. Role of Immune Check Point Inhibitors in Ovarian Cancer Treatment

Current IO is mainly based on inhibiting the PD-1/PD-L1 pathway, reinstating the immune response against tumors. Thus, the immune checkpoint blockade agents (ICIs) tested in cancer treatment are PD-1 (Pembrolizumab, Nivolumab, Dostarlimab, and Cemiplimab) or PD-L1 inhibitors (Atezolizumab, Avelumab and Durvalumab).

The efficacy of these agents depends on many factors, including PD-L1 expression, frequency of tumor infiltrating lynphocytes (TILs), neoantigen load, and tumor mutational burden [25,26].

Initial over-optimism about ICIs in OC treatment has been tempered by the disappointing results emerging in clinical trials combining these agents with standard chemotherapy or as single agents.

KEYNOTE-100 was a large phase II, single arm trial on pembrolizumab (200 mg q3w until progression of disease and for up to 2 years) enrolling 376 recurrent OC patients divided into two cohorts: 285 women who have received 1–3 prior chemotherapy lines and with a treatment-free interval of 3–12 months represented cohort A, and 91 patients with heavily pretreated disease (up to 6 prior lines) and a treatment-free interval longer than 3 months constituted cohort B. In the overall population the overall response rate (ORR) was 8% (14% and 9.9% in cohort A and B, respectively) and the disease control rate (DCR; ORR plus stabilization of disease) was 37%. Median PFS was 2.1 months in both cohorts, and median OS was 17.6 months in cohort B, while it was not reached in cohort A. The expression of PD-L1 was evaluated using CPS score: in patients with CPS <1 the ORR was 5.0%, whereas it was 10.2% and 17.1% for CPS ≥1 and CPS ≥10, respectively [27].

JAVELIN 200 (NCT02580058), a phase III, randomized trial on 566 platinum resistant OC patients, showed no advantage of the experimental arm combining pegylated liposomal doxorubicin (PLD) and avelumab vs. PLD single agent, either in terms of PFS (3.5 vs. 3.7 months for PLD and PLD-avelumb combination, respectively) nor in overall survival (13.1 vs. 15.7 months for PLD and PLD-avelumb combination, respectively) [28].

MK-3475 was a single arm phase II study enrolling 37 platinum resistant OC patients and aimed at assessing the efficacy of pembrolizumab in combination with weekly paclitaxel: the study reported an ORR of 51.4%, a disease control rate of 86.5%, with 64.5% of patients progression free at 6-month, a median PFS of 7.6 months, and a median OS of 13.4 months [29].

JAVELIN 100, a phase III randomized trial on 998 newly diagnosed OC patients testing avelumab combined with platinum-based chemotherapy, and as maintenance vs. chemotherapy alone, was prematurely terminated for futility at the pre-planned interim analysis [30].

The rationale for combining antiangiogenic agents and ICIs relies on the capability of antiangiogenetic agents to enhance T cell trafficking and infiltration into the tumor microenvironment [31]. In preclinical models, the inhibition of VEGF signaling promoted the antitumor immunity and enhanced the efficacy of immune checkpoint blockade [32], and the combination of anti-PD-L1 and anti-VEGF showed a synergistic anti-tumor effect in vivo [33].

The combination of anti-PD1 nivolumab with anti-VEGF bevacizumab was studied in a phase II study in 38 recurrent OC patients who had received up to three prior chemotherapy lines. In the overall population the ORR was 28.9% (40% vs. 16.7% in the platinum-sensitive and resistant setting, respectively); the median PFS was 9.4 months (12.1 and 7.7 months in sensitive and resistant patients, respectively). Thirty-four participants (89.5%) experienced at least one treatment-related adverse event, which was of grade 3 or higher in 23.7% of patients [34].

IMAGYN050/GOG 3015/ ENGOT-OV39, a randomized, phase III study evaluating the administration of atezolizumab/placebo in combination with carboplatin-paclitaxel-bevacizumab in 1300 newly-diagnosed, stage III-IV OC patients, showed no prognostic advantage of the experimental arm compared to the control arm (∆ PFS 1.1. months, HR 0.92 in the intent-to-treat population; ∆ PFS 2.3 months, HR 0.80 in the PD-L1-positive patients) [35]. Nevertheless, a trend favoring atezolizumab was reported in a post hoc analysis in the subgroup of patients with PD-L1 immune cells of 5% or greater, which represented about 20% of the enrolled population.

A strong rationale supports the combination of immune check points inhibitors with PARP inhibitors: HRD tumors are characterized by an elevated PD-L1 expression, thus are likely to escape immune control [36]. Moreover, the persistence of not lethal DNA defects that continuously stimulate innate immune cells to release pro-inflammatory substances probably switches from a Th1-immunity to chronic inflammation and immunosuppression [33]. PARP inhibitors, by triggering catastrophic DNA damage, especially in HRD cells, could restore a productive Th1 immune response and reset the tumor microenvironment [32,33]. In mouse models with mutations in BRCA genes, PARPi increased the mutational tumor load and TILs, and activated the interferon-mediated pathway by synergizing with ICIs, thus providing a strong rational for drug combinations [37].

In the MEDIOLA multi-cohort trial, the combination of olaparib (300 mg bid) with durvalumab (1500 mg q4w) was investigated in platinum-sensitive recurrent OC patients who had a known or suspected deleterious germline BRCA1/2 mutation.

The ORR was 71.9% (with 7 CRs), with a median duration of response (DOR) of 10.2 months and a 28-week DCR of 65.6%; median PFS was 11.1 months and median OS was not reached, with 87.0% of patients alive at 24 months [38].

The TOPACIO/KEYNOTE-162 trial explored the combination of niraparib and pembrolizumab in women with advanced or metastatic triple-negative breast cancer (TNBC) or recurrent OC, irrespective of BRCA mutation status. Among the OC cohort (60 patients), the ORR was 18%, including three confirmed CRs and eight PRs, with a DCR of 65% and a median DOR not reached (range, 4.2 to ≥14.5 months); notably the ORR was consistent across the subgroup analysis based on platinum sensitivity, previous bevacizumab treatment, and tumor BRCA and/or HRD biomarker status [39].

Many ongoing clinical trials are focused on exploring the advantages of the combinations of immune check point inhibitors with PARP and/or antiangiogenetic agents in newly diagnosed or recurrent OC in combination with chemotherapy, and/or as maintenance (see Table S1) [40].

Finally, according to what emerged in other neoplasms, and given the redundant and cross talk mechanism of immune control, the dual immune checkpoint blockade was explored as a strategy to improve PD1-PDL1 blocking efficacy and overcome resistance [41].

The NRG-GY003 trial was a randomized phase II study on 100 recurrent OC patients aimed at comparing nivolumab versus nivolumab and ipilimumab. The combination of nivolumab and ipilimumab resulted in superior ORR (12.2% vs. 34%) and longer, albeit limited, PFS (2 months vs. 3.9 months) compared to nivolumab alone [42].

In this scenario, the prolonged response achieved by about 50% of patients and the manageable safety profile of IM warrants, in our opinion, further investigation. Probably the best setting to test IO is in newly diagnosed disease, counting on a non-exhausted immune system. Moreover, clinical data suggest that a better selection of patients according to PD1/PD-L1 expression may identify a subgroup of patients who may benefit more from the strategy. Finally, recent data suggest that the constitutional OC activated pathway, such as TGF-beta, may be involved in next generation ICI with a double block in TGF-beta and PD-l1, and may overcame resistance to IO.

## 5. Conclusions

Targeted therapies are a supporting pillar in OC treatment strategy. In particular, bevacizumab and PARP inhibitors have transformed OC from a “killer” to a chronic disease with options for management. Maintenance treatment is currently able to delay disease progression with manageable toxicity but it is less clear what impact it has on the efficacy of subsequent therapies. The best treatment sequence (combination vs. sequencing of antiangiogenic agents and PARPi, and what is the best sequence) as well as the optimal patient selection for each treatment remain key objectives of clinical research, with the aim of optimizing the available therapeutic options for OC patients.

Finally, despite disappointing results in many settings of OC disease, as single agents and in combination with antiangiogenic agents, IO remains an area of great interest in OC clinical research.

The unavailability of a reliable predictive biomarker of response and, as a consequence, a bias in patient selection may be addressed in the coming years, improving IO therapeutic performances in OC. Our expectations and hopes, in particular, are that the ongoing trials combining ICIs with PARP inhibitors will clarify the role, if any, of immunotherapy in OC treatment.

## Supplementary Materials

The following are available online at https://www.mdpi.com/2072-6694/13/6/1298/s1, Table S1: Ongoing clinical trials of immunotherapy combined with other drugs in ovarian cancer.
