# Supplementary material for: Ovarian Cancer Treatments Strategy: Focus on PARP Inhibitors and Immune Check Point Inhibitors
Source: Cancers (Basel). 2021 Mar 15;13(6):1298. doi: 10.3390/cancers13061298 (PMC7999042; doi:10.3390/cancers13061298)
Supplement: Supplementary file 1 [file cancers-13-01298-s001.pdf]

Table S1. Ongoing clinical trials of immunotherapy combined with other drugs in ovarian cancer.

Trial	Phase	Setting	Treatment	Start Date-Estimated Study Completion Date	Target Enrollment	Primary Outcome
AGO DUO (NCT03737643)	III	Advanced 1L	platinum-based chemotherapy plus bevacizumab ± durvalumab followed by maintenance bevacizumab ± durvalumab ± olaparib	January 4, 2019>> November 14, 2025	1254	PFS
ENGOT OV 43 (NCT03740165)	III	Advanced 1L	paclitaxel/carboplatin in combination with ± bevacizumab (investigator choice) ± pembrolizumab followed by maintenance ± bevacizumab ± pembrolizumab ± olaparib	December 18, 2018>>August 8, 2025	1086	PFS<br>OS
FIRST (NCT03602859)	III	Advanced 1L	carboplatin+paclitaxel plus bevacizumab in combination with ± TSR-042 followed by ± Niraparib ± TSR-042 maintenance therapy	October 11, 2018>> July 22, 2026	1228	PFS
ATHENA trial (NCT03522246) *	III	Advanced 1L	platinum-based chemotherapy followed by maintenance ± rucaparib in combination with ± nivolumab	May 14, 2018>> December 30, 2030	1000	PFS
MITO 28 (NCT03410784)	II	Advanced 1L	Carboplatin-paclitaxel + pembrolizumab followed by maintenance pembrolizumab	April 2018>> December 2020	72	
N-Dur (NCT02726997)	I/II	Advanced 1L	Carboplatin-paclitaxel + durvalumab	July 6, 2016>> July 30, 2022	35	Pharmacodynamic Changes
NCT03353831	III	1st or 2nd relapse within 6 months after platinum based chemotherapy	platinum-based chemotherapy plus bevacizumab in combination with ± atezolizumab followed by maintenance bevacizumab ± atezolizumab	September 11, 2018>> July 1, 2022	664	OS<br>PFS
NRG-GY009 (NCT02839707)	II/III	platinum-resistant recurrent OC	pegylated liposomal doxorubicin hydrochlorid ± bevacizumab ± atezolizumab	June 23, 2017>> July 12, 2027	488	Dose limiting toxicities<br>PFS<br>OS
ATALANTE (NCT02891824)	III	platinum-sensitive recurrent OC	platinum-based chemotherapy plus bevacizumab in combination with ± atezolizumab followed by maintenance bevacizumab ± atezolizumab	September 22, 2016>> September 2023	614	PFS
NCT02659384	II	advanced recurrent platinum-resistant OC patients	Bevacizumab monotherapy ± atezolizumab ± acetylsalicylic acid	December 2016>> February 2023	122	PFS
ANITA (NCT03598270)	II	recurrent platinum-sensitive OC patients (up to 2 prior lines)	platinum-based chemotherapy ± atezolizumab followed by maintenance niraparib ± atezolizumab	November 21, 2018>> May 2024	414	PFS
AGO-OVAR 2.29 (NCT03353831)	III	recurrent platinum-resistant OC patients (up to 3 prior lines)	platinum-based chemotherapy plus bevacizumab in combination	September 11, 2018>> July 1, 2022	664	OS<br>PFS

			with ± atezolizumab followed by maintenance bevacizumab ± atezolizumab			
NCT0248440	I**/II	advanced solid tumors including persistent advanced or platinum-resistant recurrent OC (2 prior platinum-containing regimens)	durvalumab in combination with ± olaparib ± cediranib	June 29, 2015>> December 29, 2022	384	Ph I: recommended phase II dose<br>Ph II: ORR
NCT02431559	I/II	recurrent, platinum-resistant OC	Motolimod + Doxorubicin + Durvalumab	December 2, 2015>> June, 2020	53	• Adverse Events<br>• PFS
NCT02571725	I/II	recurrent BRCA mutation-associated OC	Olaparib + Tremelimumab	February 2016>> February 2022	50	• Recommended Phase 2 Dose<br>• ORR
NCT02853318	II	recurrent epithelial ovarian, fallopian tube, or primary peritoneal cancer	pembrolizumab + bevacizumab + oral metronomic cyclophosphamide	September 1, 2016>> August 3, 2021	40	• Adverse Events<br>• PFS
NCT02440425	II	platinum resistant recurrent OC	dose dense (weekly) paclitaxel + pembrolizumab	October 20, 2015>> September 2023	43	• PFS<br>• Adverse Events
EUDRA CT 2020-000146-33 NItCHE (MITO 33)	III	platinum resistant recurrent OC	Niraparib+Dostarlimab vs chemotherapy	December 2020>>December 2024	427	• OS
EUDRA CT 2019-004365-41 (MITO 27)	II	platinum resistant recurrent OC, CPS >1	pembrolizumab	2021>>2023	100	• OS

* completion of the target enrollment as first-line maintenance treatment in combination with nivolumab has been just announced [1] ** Positive interim results from the phase I phase on OC patients were published [2].

References

1. Clovis Oncology Announces Completion of Target Enrollment in the ATHENA trial, a Phase 3 Maintenance Treatment Study in Front-Line, Newly-Diagnosed Advanced Ovarian Cancer. News Release. *Clovis Oncol.* Available online: https://bit.ly/2Nbn7a4 (accessed on 30 June 2020).
2. Zimmer, A.S.; Nichols, E.; Cimino-Mathews, A.; Peer, C.; Cao, L.; Lee, M.J.; Kohn, E.C.; Annunziata, C.M.; Lipkowitz, S.; Trepel, J.B.; et al. A phase I study of the PD-L1 inhibitor, durvalumab, in combination with a PARP inhibitor, olaparib, and a VEGFR1-3 inhibitor, cediranib, in recurrent women's cancers with biomarker analyses. *J. Immunother. Cancer* **2019**, *7*, 197, doi:10.1186/s40425-019-0680-3.
